# Inhibition of LONP1 in prostate cancer: bibliometrics-guided target screening and AI-driven antibody design

**DOI:** 10.1097/JS9.0000000000004705

**Published:** 2026-01-19

**Authors:** Jiawei Pan, Yuan Zhang, Anqi Yang, Linglong Jiang, Yuwei Shen, Yangyang Sun, Jundong Zhu, Zhen Chen, Min Fan, Jian Shi

**Affiliations:** Department of Urology, The Third Affiliated Hospital of Soochow University, Changzhou, China

**Keywords:** bibliometric, GeoBiologics, LONP1, mitochondria, prostate cancer

## Abstract

**Background:**

Prostate cancer remains one of the most common malignant tumors among men worldwide, with its incidence showing a continuous global increase. In recent years, mitochondria-targeted therapeutic strategies have emerged as a prominent research focus in oncology. However, a systematic analysis of the research trends concerning mitochondria in prostate cancer treatment is currently lacking. This study employed bibliometric methods to conduct a comprehensive analysis of the dynamic progress in mitochondria-related prostate cancer research, ascertain its significant role, and identify potential mitochondria-targeted therapeutic targets. Furthermore, using computer-aided methods, we designed and optimized a specific antibody, providing a candidate strategy for prostate cancer control.

**Methods:**

This study utilized the Web of Science Core Collection database (2015–2023) to perform visual analysis of country-keyword network relationships using CiteSpace and the Bibliometric Online Analysis Platform. Target screening was conducted by integrating bioinformatics and research intelligent agents. Subsequently, inhibitory antibodies were designed and screened based on GeoBiologics, followed by systematic *in vitro* evaluation of their purity, antigen-antibody affinity, conformational stability, colloidal stability, and enzymatic inhibitory activity.

**Results:**

The role of mitochondria in prostate cancer has garnered significant attention. Research trends have shifted from fundamental mechanisms to addressing drug resistance, developing novel delivery systems, and exploring combination therapies, highlighting mitochondria as a promising target for clinical intervention. Lon Peptidase 1(LONP1) is closely associated with mitochondrial homeostasis and prostate cancer progression. Antibody_82-M1 effectively blocks the ATP-binding site of LONP1, demonstrating high affinity, favorable stability, a high degree of humanization, and excellent drug-like properties, indicating strong potential for clinical translation.

**Conclusion:**

The designed LONP1 inhibitory antibody offers a novel strategy for prostate cancer treatment. The proposed workflow – “bibliometric guidance – research intelligent agent screening – bioinformatics support” – proves beneficial for enhancing AI-driven scientific research and optimizing the screening of therapeutic targets for diseases.

## Introduction

According to data from the World Health Organization, prostate cancer is the second most prevalent malignant tumor among men worldwide^[1]^. A report published in The Lancet forecasts that the number of prostate cancer cases globally will rise to 2.9 million by 2040^[2]^. With the ongoing aging of the population, the incidence and mortality rates of prostate cancer in men continue to escalate, rendering it a significant global public health concern and imposing an increasingly substantial burden on healthcare systems worldwide^[3]^. Current clinical treatments for prostate cancer primarily encompass prostatectomy, radiation therapy, and hormone therapy; however, these methods exhibit certain limitations. A notable proportion of patients with advanced-stage disease progress to castration-resistant prostate cancer (CRPC), and the existing treatment options for CRPC are often associated with drug resistance and limited efficacy. Consequently, there is an urgent necessity to investigate new therapeutic strategies^[4–6]^.

A substantial body of evidence indicates a close relationship between mitochondrial function and the initiation and progression of tumors^[7,8]^. However, systematic summarization of this research field in the context of prostate cancer remains relatively limited. To address this gap, this study employed bibliometric methods, utilizing tools such as CiteSpace, to conduct a visual analysis of research themes related to mitochondria and prostate cancer. This was achieved through literature coupling and keyword co-occurrence mapping, aiming to identify research hotspots, developmental trends, and potential frontiers in the field.

In recent years, artificial intelligence scientists have demonstrated multi-dimensional and profound potential in the field of drug discovery. Their impact has evolved from merely enhancing the efficiency of individual tools to systematically reshaping the structure of research systems and working paradigms. AI not only autonomously uncovers patterns and correlations from massive datasets that are difficult for humans to detect – thereby supplementing and even partially replacing traditional hypothesis-driven research pathways – but more importantly, it breaks down barriers between traditional disciplines by integrating diverse knowledge^[9,10]^. This fosters interdisciplinary convergence and facilitates the “intelligent emergence” of new knowledge. Against this backdrop, the rise of general biomedical AI agents, exemplified by Biomni, marks a significant transition in AI’s role in biomedical research – from a “tool user” to a semi-autonomous “research participant.” Biomni integrates 150 specialized biological tools and 59 comprehensive biomedical databases, exhibiting strong zero-shot generalization capabilities that enable it to autonomously execute complex multimodal biomedical analyses. In this study, we utilized Biomni to assist in identifying the key mitochondrial regulator LONP1.

Furthermore, with the recent robust emergence of antibody-drug conjugates (ADCs, AOCs, ACCs, and ABCs), generative AI strategies have been widely adopted by biopharmaceutical companies due to their unique advantages of high throughput and efficiency. Such platforms systematically enable *de novo* antibody generation, affinity maturation, and drug-likeness optimization, significantly shortening the discovery and optimization cycles of candidate molecules^[11]^. In this study, we employed BioGeometry’s GeoBiologics platform, a representative tool in this cutting-edge direction. Built on the generative AI model GeoFlow, this platform specializes in antibody design and, through innovations in geometric deep learning architectures, achieves a success rate of up to 80% in critical tasks such as antigen-antibody docking. Its core strength lies in its ability to learn from vast datasets while simultaneously optimizing multiple key attributes of antibodies, demonstrating powerful capabilities in de novo antibody design, affinity maturation, and drug-likeness optimization^[12]^. Using this platform, we designed a humanized inhibitory antibody targeting LONP1 and, after sequence optimization, systematically validated its biological efficacy through *in vitro* experiments. This study adheres to the TITAN Guidelines 2025 for the declaration and use of AI in research^[13]^.

This study not only provides new insights and candidate strategies for prostate cancer treatment but also represents a successful implementation of a human-AI collaborative research paradigm.

## Materials and methods

### Data collection and pre-processing

The data for this study were sourced from the Science Citation Index Expanded within the Web of Science Core Collection. To systematically retrieve relevant literature, two separate search strategies were constructed:

Search Strategy 1: TS = (“mitochondria” OR “mitophagy”) AND (“prostatic cancer” OR “prostatic carcinoma” OR “prostatic adenocarcinoma” OR “prostate tumor” OR “prostatic malignancy” OR “prostatic neoplasm” OR “radical prostatectomy”)

**HIGHLIGHTS**

- This study innovatively proposes a “bibliometrics guidance – research intelligent agent screening – bioinformatics support” paradigm for identifying therapeutic targets in prostate cancer, ultimately pinpointing LONP1.
- Targeting LONP1 as a druggable mitochondrial inhibitor, we employed GeoBiologics to design a novel humanized antibody (Antibody_82) that specifically blocks the ATP-binding site of LONP1.
- Validated by *in vitro* experiments, this antibody demonstrates excellent binding affinity, favorable stability, and potent enzymatic inhibition, presenting a highly translatable candidate for prostate cancer treatment.

Search Strategy 2: TS = (“mitochondria” OR “mitophagy”) AND (“LONP1” OR “lon protease homolog, mitochondrial”).

The search was limited to publications from 2015 to 2023, with language restricted to English. Document types included articles, online publications, and review articles. Ultimately, 452 and 194 relevant publications were retrieved using Strategy 1 and Strategy 2, respectively. All records were exported in plain text format for subsequent citation and analysis. The entire process of literature collection, screening, and data handling strictly adhered to the systematic review guidelines recommended by the PRISMA statement^[14]^.

### Analysis and visualization

For this study, we primarily utilized CiteSpace (version 5.8.R2) and the Bibliometric Online Analysis Platform (http://bibliometric.com/) to identify network characteristics such as co-cited references, keywords, and countries, thereby visualizing the research findings. CiteSpace was employed specifically to perform a co-occurrence analysis of keywords. Additionally, the online bibliometric platform was used to conduct co-authorship analysis at the national level and to analyze publication trends.

### Application of intelligent agents and bioinformatics analysis

The large language model-driven agent, Biomni, was employed to integrate mitochondrial gene sets from the MitoProteome database with all literature data from the past 3 years concerning mitochondria and cancer. The top 20 relevant genes were initially screened based on their frequency of occurrence. Subsequently, UALCAN (https://ualcan.path.uab.edu/analysis.html) was utilized to examine the expression differences of these genes between cancerous and normal tissues, while Biomni assisted in analyzing their drug-gability potential. Enrichr-KG (https://maayanlab.cloud/enrichr-kg) was applied to investigate the biological processes associated with the LONP1 gene. Furthermore, GSCA (https://guolab.wchscu.cn/GSCA/#/) was used to analyze the correlation between drug sensitivity and LONP1 mRNA expression, as well as the Spearman correlation between the LONP1 gene and prostate cancer-related pathways.

### Antibody design, optimization, and wet-lab validation

Utilizing BioGeometry’s GeoBiologics platform (https://geobiologics-cn.biogeom.com/index/trial), specifically its Targeted complementarity-determining region (CDR) Library Design module, we constructed a targeted CDR library for LONP1. During the design process, the epitope and antibody framework were clearly defined. By referencing the UniProt database, we identified the ATP-binding site of LONP1 (P36776) located at residues 523–530 on chain C, along with potential antigenic regions. Ultimately, the segment from residues 421–558 on chain C was selected as the antibody-binding site, and its structural file (PDB: 7KSL) was uploaded into the GeoFlowDesign model. For the antibody framework, we chose the trastuzumab monoclonal antibody (PDB: 1N8Z), which is widely used in constructing novel antibody candidates. The GeoFlowDesign model automatically generated 100 candidate sequences, each of which underwent preliminary evaluation for affinity and specificity in a simulated environment. Four high-performing antibody sequences were selected, and by comparing various scoring metrics – such as pTM, ipTM, wpTM, and All pLDDT – the optimal antibody sequence, Antibody_82, was identified.

For Antibody_82, systematic structural optimization and humanization were conducted. Initially, the CDRs of both the heavy chain (HC) and light chain (LC) were identified and annotated according to IMGT standards. Molecular docking between the antibody and target was executed using HDOCK, with the conformation exhibiting the highest score selected as the initial complex structure. Utilizing GROMACS 2024 software and the AMBER99SB force field, a molecular dynamics (MDs) workflow was implemented, which included energy minimization, system heating to 300 K, NPT equilibration, and a 50 ns production simulation. The binding free energy was calculated using gmx_MMPBSA, and per-residue energy decomposition within the CDR regions was performed to identify unfavorable binding residues (ΔG > 0.3 kcal/mol) as targets for mutation. Virtual saturation mutation (excluding Cys, totaling 18 amino acids) was conducted on these residues employing the CHARMm force field. The ΔΔG of each mutant was evaluated, leading to the selection of a combination of mutations that significantly reduced ΔΔG and enhanced hydrophobicity – specifically HC_Lys30Glu, HC_Asp104Trp, and LC_Thr95Trp. Mutant models were constructed using PyMOL and underwent further dynamics simulations and binding free energy validation. Subsequently, BioPhi was utilized for humanization design to improve germline gene compatibility and OASis identity recognition rate. Finally, the developability of the humanized antibody – including hydrophobicity and charge distribution – was assessed using Therapeutic Antibody Profiler, and T-cell epitopes were predicted via the IEDB-NetMHCIIPan algorithm to evaluate immunogenicity risk.

In wet-lab experiments, Antibody_82-M1 was transiently expressed in HEK293 cells. After 5 days of culture, the supernatant was harvested and purified using Protein A affinity chromatography (POROS MabCapture A Select) under neutral pH conditions. Elution was performed with low-pH glycine-HCl (pH 3.5), followed by immediate neutralization with Tris-HCl (pH 9.0). The final buffer was exchanged to 20 mM His (pH 6.0) via ultrafiltration. Antibody purity was assessed using HPLC-SEC with an AdvanceBio SEC 300 Å column, employing a mobile phase of 2× PBS and a detection wavelength of 214 nm, in addition to CE-SEC. Affinity measurements were conducted on a Biacore 8 K system, where antibodies were captured on a Protein G chip, and binding kinetics were assessed using gradient concentrations of LONP1 as the analyte in 1 × HBS-EP buffer. Binding and dissociation were monitored and fitted to a 1:1 binding model. Conformational stability was evaluated by differential scanning fluorometry, where samples in 20 mM His (pH 6.0) were heated from 25°C to 95°C at a rate of 1°C per minute, and denaturation temperature was analyzed based on intrinsic fluorescence changes. Colloidal stability was monitored via dynamic light scattering and static light scattering under the same heating conditions to evaluate aggregation propensity.

Subsequently, we evaluated the inhibitory effect of the antibody on LONP1 protease activity through an *in vitro* enzymatic assay. The reaction system contained 0.2 nM LONP1, 1 nM ATP, and the substrate protein Mitochondrial Pyruvate Carrier 1 (MPC1). It is noteworthy that LONP1 directly targets MPC1 – a key metabolic protein in glycolysis – enhancing its degradation, suppressing the tricarboxylic acid cycle, and ultimately promoting prostate cancer progression^[15]^. Antibody_82 was serially diluted in a three-fold gradient starting from 4 nM (totaling seven concentrations) and incubated with the enzyme system at 37°C for 30 minutes. The residual amount of undigested MPC1 was measured using ELISA, and the inhibition rate was calculated using the formula: 1 − (residual MPC1/initial MPC1).

## Results

### Bibliometric analysis highlights mitochondria as a key therapeutic target in prostate cancer

**Figure 1.**
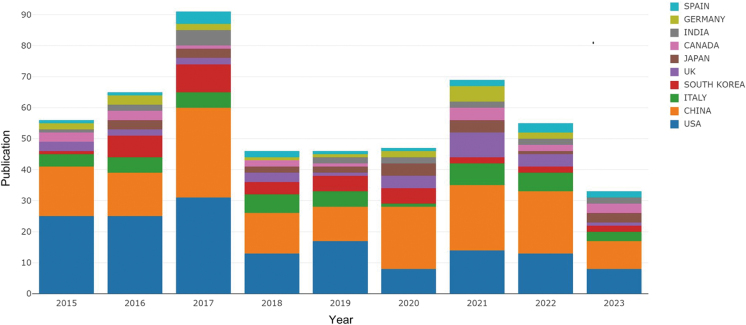
Annual publication and the contribution characteristics of each country.

We collected a total of 452 research articles related to mitochondria and prostate cancer. These publications have generated significant impact in the academic community, with a cumulative total of 17 774 citations, reflecting the importance and attention devoted to this field of research. As shown in Figure 1, the annual number of publications increased markedly between 2013 and 2017, followed by a decline after 2018. Specifically, from 2018 to 2020, the publication output plateaued; it then rose again in 2021, with a slight decrease observed in 2023.

**Figure 2.**
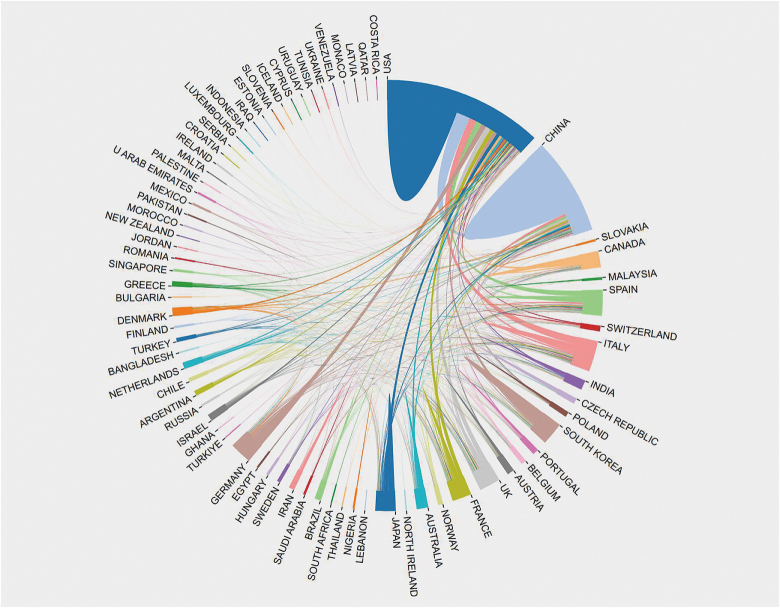
Global collaborative networks among countries.

A total of 77 countries contributed to research in the field of mitochondria and prostate cancer in this study. Figure 1 displays the annual publication output trends from 2015 to 2023 for the top 10 most productive countries. The United States held the leading position in publication volume until 2020, when it was overtaken by China. Figure 2 illustrates the collaborative relationships between different countries, where thicker lines indicate stronger collaborative ties. The United States maintained the most frequent collaborations with other countries in this field, followed by China. The collaborative relationship between the United States and China was particularly strong.

**Figure 3.**
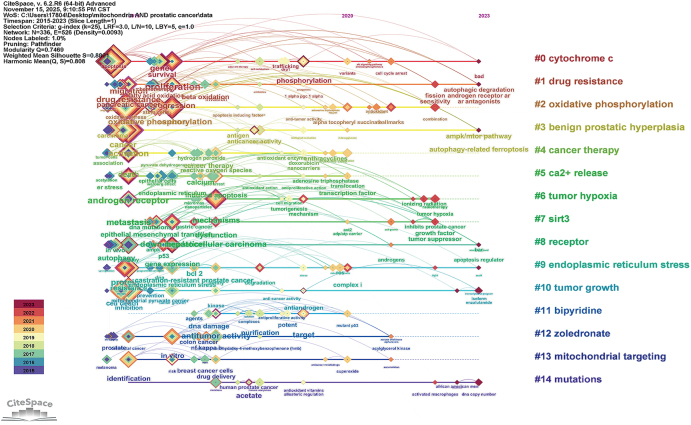
Keyword timezone map of mitochondria and prostate cancer.

We also constructed a timeline visualization of co-cited keywords to better understand the evolution of research themes. In Figure 3, nodes along the timeline are color-coded by their publication years. The analysis reveals a clear chronological progression: research initially focused on fundamental mitochondrial functions and tumor phenotypes (2015–2017), with prominent keywords including “aerobic glycolysis,” “oxidative phosphorylation,” “proliferation,” and “migration,” highlighting investigations into how mitochondrial metabolic reprogramming drives prostate cancer progression. The focus subsequently transitioned to molecular mechanism exploration (2018–2020), emphasized by keywords such as “DNA mutations” and “phosphorylation.” The most recent phase (2021–2023): Focusing on clinical translation and combination therapy, key terms such as “drug resistance,” “tumor hypoxia,” “nanocarriers,” and “combination therapy” emerged as new hotspots. This reflects the shift of research from basic mechanisms toward addressing clinical drug resistance, developing novel drug delivery systems, and formulating combination therapy strategies, demonstrating that studies on mitochondrial mechanisms in prostate cancer have moved toward mitochondria-targeted “clinical applications.”

### LONP1 identified as a key therapeutic target

**Figure 4.**
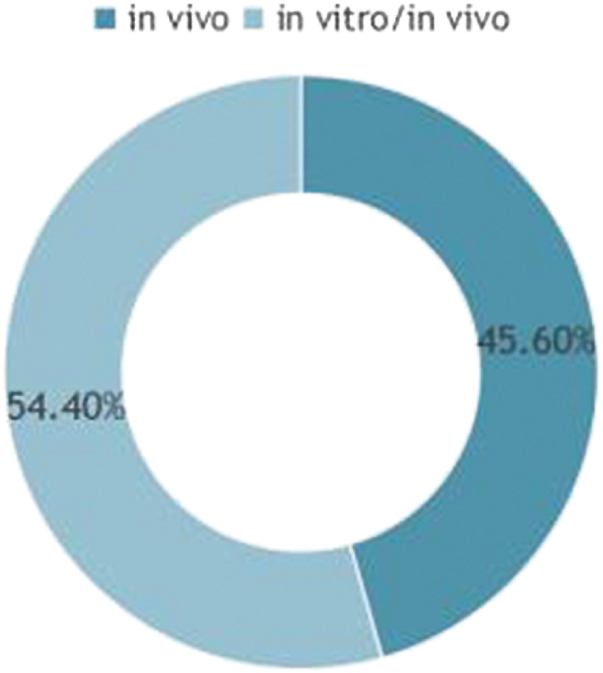
Statistics of experimental methods.

Furthermore, we further refined our selection to 241 articles involving experimental studies, among which 110 focused on *in vivo* experiments and 131 encompassed both *in vivo* and *in vitro* experiments (Fig. 4). Among these studies, 152 concentrated on mitochondrial-targeted therapies, with 29 involving combination treatment strategies. Notably, nearly one-third of the studies were closely related to mitochondrial-targeted therapy, which underscores the significance of this area in current research.

Based on these findings, to identify suitable mitochondrial targets for intervention, we first downloaded a human mitochondrial gene dataset from the MitoProteome database. We then retrieved all literature published in the past 3 years using the keywords “mitochondria” and “cancer” and uploaded the collected data into the large language model-driven Biomni platform for automated screening and comparison. Finally, the top 20 genes by frequency of occurrence were listed (Supplemental Digital Content Table S1, available at: http://links.lww.com/JS9/G713). Notably, genes such as PARKIN, DRP1, PINK1, BCL2, NRF2, MFN2, OPA1, TFAM, SIRT3, BNIP3, MFN1, FIS1, TOM20, UCP1, BNIP3L, MFF, and NRF1 were found to be downregulated or exhibited no significant differences in prostate cancer. Directly targeting these genes for activation or functional restoration poses considerable challenges, as upregulation in the context of pre-existing expression deficiencies is often inefficient and susceptible to compensatory pathways or off-target effects, which ultimately results in limited therapeutic efficacy and low translational potential^[16]^. In contrast, BAX, VDAC1, HSP60, and LONP1 were significantly upregulated in prostate cancer. BAX functions as a potent tumor suppressor and serves as a core effector protein within apoptosis and tumor suppression networks; thus, inhibiting its activity may promote tumor growth^[17]^. VDAC1 is associated with the progression and aggressiveness of prostate cancer; however, there is no significant correlation between the expression level of the VDAC1 gene and the prognostic survival rate of prostate Prostate Adenocarcinoma (PRAD) patients. Furthermore, it has been reported that in cholangiocarcinoma, low VDAC1 expression is positively correlated with aggressive phenotypes, lymph node metastasis, and reduced patient survival. Additionally, the substantial cardiotoxicity of VDAC1 inhibitors limits the drug’s safety window, further complicating targeted therapy^[18]^. HSP60 is a crucial chaperone that plays a vital role in maintaining cellular function. However, its extensive and intricate regulatory roles render it unpredictable and unsuitable as a therapeutic target^[19,20]^. Conversely, Cytochrome C (CYTC) is a pivotal molecule involved in apoptosis; however, it does not possess a suitable interface for small-molecule binding, which complicates direct targeting. Additionally, its functionality is contingent upon protein interactions, further categorizing it as undruggable^[21]^.

**Figure 5.**
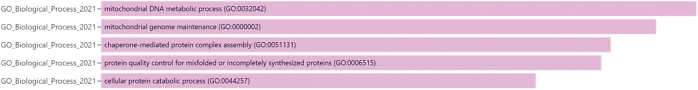
Biological processes associated with the LONP1 gene.

**Figure 6.**
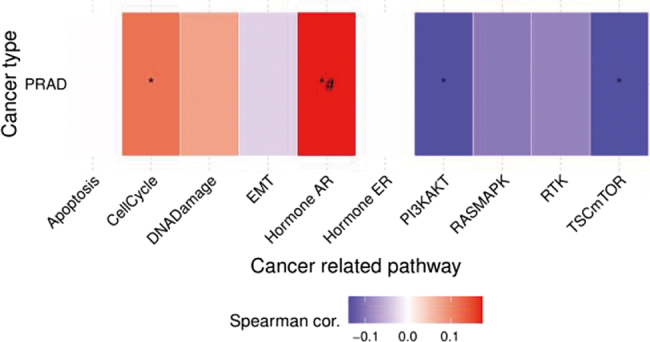
Spearman correlation of LONP1 gene with prostate cancer-related pathways.

**Figure 7.**
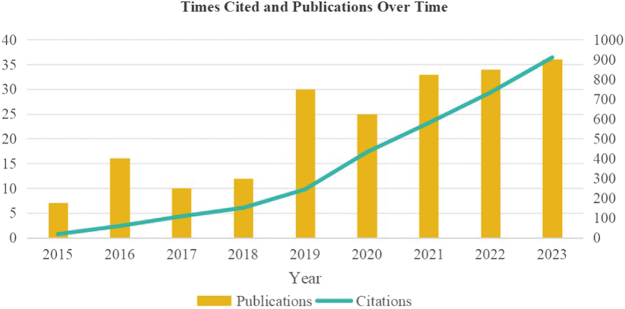
Annual publication count and citation count for research on LonP1 and mitochondria.

**Figure 8.**
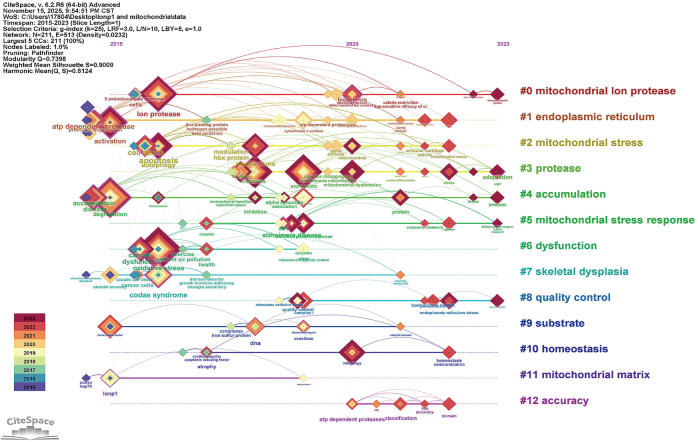
Keyword timezone map of LONP1 and mitochondria.

In contrast, although LONP1 ranked 18th in literature frequency, its biological functions are well defined. As an ATP-dependent protease located in the mitochondrial matrix, its structural domains primarily include an N-terminal domain, an AAA + ATPase domain, and a proteolytic domain, all playing critical roles in maintaining its proteolytic activity and mitochondrial function, with the ATPase domain serving as the rate-limiting site for its activity^[22]^. To further systematically elaborate on the biological background of LONP1 and its potential role in PRAD, we first performed Gene Ontology functional enrichment analysis. The results revealed that LONP1 is significantly involved in regulating mitochondrial DNA replication and transcription processes, and influences the stability of related regulatory factors, indicating its crucial role in maintaining mitochondrial quality homeostasis (Fig. 5). Building on these findings, we further investigated pathway-level associations of LONP1 in PRAD using the GSCA database. The analysis demonstrated that LONP1 is closely linked to several core tumor-related biological processes: it shows a positive correlation with cell cycle progression and androgen receptor signaling, while exhibiting negative correlations with both the PI3K/AKT and TSC/mTOR signaling pathways (Fig. 6). Existing clinical analyses further demonstrate that high LONP1 expression is significantly and positively associated with elevated Gleason scores, advanced pathological stages, epithelial-mesenchymal transition progression, and adverse clinical outcomes in PRAD^[23]^. However, bioinformatic analysis can only reveal correlations of LONP1 in PRAD without reflecting research attention. To further evaluate its research value, we conducted a bibliometric analysis using “LONP1” and “mitochondria” as keywords. The results showed a steady increase in publications on LONP1 and mitochondria from 2015 to 2023 (Fig. 7). Keyword co-occurrence analysis (Fig. 8) demonstrated that cancer remains the primary research focus, while early attention on Alzheimer’s disease and skeletal disorders has gradually declined. The research evolution progressed from early focuses on apoptosis, autophagy, and endoplasmic reticulum stress to current interests in mitochondrial homeostasis/communication and mitophagy. Additionally, mitochondrial oxidative processes and hydrolysis mechanisms have remained consistently important. These findings align with the mechanistic role of LONP1 in PRAD revealed by bioinformatics and the key regulatory nodes of mitochondria in prostate cancer treatment identified in our initial bibliometric analysis. Furthermore, animal studies have demonstrated that LONP1 inhibition through either siRNA interference or small molecule drugs (CDDO-Me) effectively suppresses prostate cancer growth^[23]^. Collectively, these results suggest that LONP1 promotes prostate cancer progression through mitochondrial mechanisms, positioning it as a highly valuable target for drug development.

**Figure 9.**
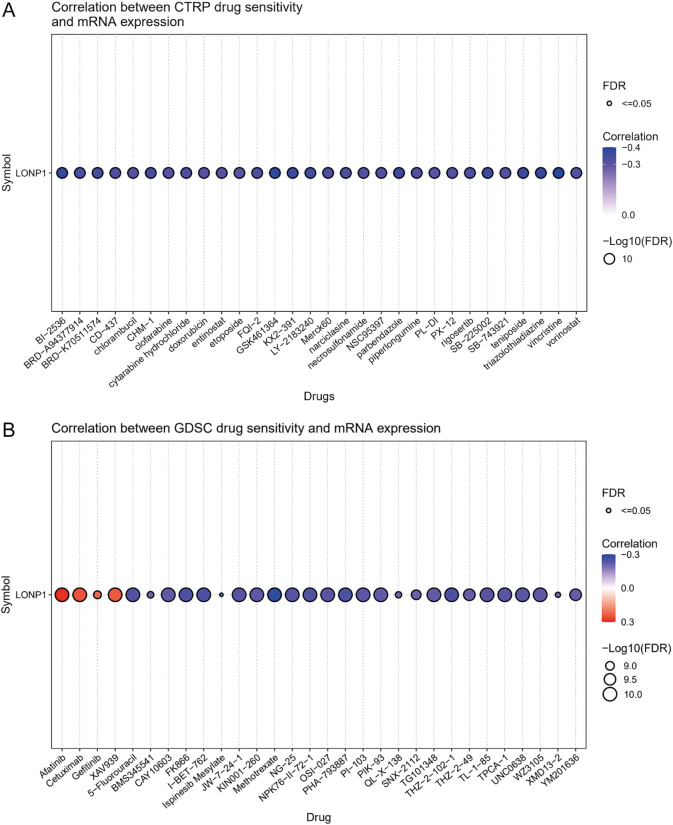
(A and B) Correlation analysis between GDSC drug sensitivity and LONP1 mRNA expression.

Subsequently, to explore intervention strategies targeting LONP1, we first investigated the relationship between LONP1 expression and anticancer drug sensitivity using the GDSC database. As shown in Figures 9A, 9B, LONP1 expression demonstrated significant negative correlations with sensitivity to several agents, including Vincristine, GSK461364 (Parthenate), Methotrexate, and THZ-2-102-1. However, these small-molecule drugs present considerable limitations in clinical applications. Primarily consisting of conventional chemotherapeutic agents, they often lead to acquired resistance in patients and exhibit poor specificity. Furthermore, they are frequently associated with various adverse effects – such as bone marrow suppression, gastrointestinal reactions, increased cardiovascular risks, hepatotoxicity, and nephrotoxicity – which substantially compromise patients’ quality of life and complicate clinical management^[24–26]^. Even more recently reported small-molecule LONP1 inhibitors like CDDO-Me, which function through Michael adduct formation, are susceptible to intracellular redox conditions and may undergo cross-reactions with cysteine-containing proteins, resulting in considerable off-target risks. These inherent drawbacks significantly hinder their clinical translation and therapeutic potential^[27]^.

In contrast, targeted therapeutic antibodies have demonstrated distinct advantages in cancer treatment, characterized by high specificity, rapid onset of action, extended half-life, and minimal off-target risks. This profile suggests superior application potential compared to both small-molecule drugs and gene-targeting therapies^[28]^.

### A high-affinity anti-LONP1 antibody was successfully designed and optimized

Currently, no therapeutic antibodies targeting LONP1 have received approval. To address this unmet medical need, we designed and screened a humanized antibody against LONP1 using the GeoBiologics platform, ultimately identifying the candidate sequence Antibody_82. This antibody exhibited excellent performance across multiple structure-function metrics, with the following scores: identifier score of 0.644, pTM of 0.462, ipTM of 0.498, and wpTM of 83.094. In terms of pLDDT metrics, we observed an All pLDDT score of 88.555, a Binder pLDDT score of 80.558, and a Target pLDDT score of 4.264. For pAE metrics, the Binder pAE score was 11.062, the Target pAE score was 19.46, and the Interaction pAE score was 0.88. Furthermore, the Binder aligned lRMSD was measured at 76.936. Collectively, these results indicate a strong affinity and specificity.

To further evaluate its clinical translatability, we assessed the humanness of the antibody using the Humanness Prediction module. Antibody_82 exhibited a moderate humanness score of 57.9%, alongside a high germline compatibility of 85.3%. Subsequent developability predictions conducted through the Developability Prediction module indicated a SEC-HPLC purity of 95.887% and a yield of 477.146, positioning it among the highest-ranking candidate molecules in its class, thereby demonstrating favorable developability and production potential.

**Figure 10.**
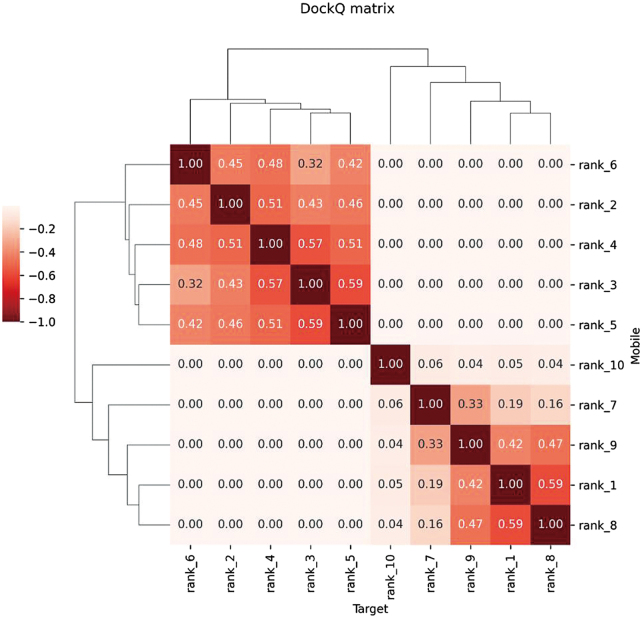
Clustermap of DockQ scores for predicted structures.

Additionally, we conducted antigen-antibody molecular docking utilizing the Antibody-Antigen Docking module. The binding mode and quality between LONP1 and Antibody_82 were quantitatively assessed using the DockQ scoring system, which helped validate the rationality and stability of the binding conformation. Figure 10 presents a clustering heatmap of the predicted structures, demonstrating high similarity among the predictions and a close approximation to the native structure during docking.

Table 1 presents the DockQ score, final energy (E_final), and buried surface area (Buried ASA) for each docked structure. The average DockQ score for the predicted structures was 0.29, with an antigen-antibody binding energy of −25 801.87 cal/mol. These results suggest that the predicted structures exhibit a certain level of stability in the docking conformation; however, they still do not meet the desired benchmark. Consequently, we undertook additional sequence optimization to enhance the binding performance.

**Table 1:** Ranking of docking poses based on scoring and energetics.

Rank	Score	Efinal.	Buried ASA
1	0.3	−24 774.61	1768.85
2	0.3	−27 402.56	1673.87
3	0.3	−26 590.96	1598.72
4	0.3	−24 863.89	1443.74
5	0.29	−28 040.66	1053.32
6	0.28	−24 478.79	1912.87
7	0.28	−23 385.86	1820.72
8	0.28	−25 729.61	1624.82
9	0.28	−26 998.67	1570.27
10	0.28	−26 937.49	1813.71

**Table 2:** Decomposition results of antibody-antigen binding free energy.

Energy component	Average	SD
AVDWAALS	−101.21	7.94
AEEL	−272.74	51.75
ΔEGB	346.12	50.82
ESURF	−13.57	1.32
ΔGGAS	−373.95	54.69
AGSOLV	332.55	49.88
ΔTOTAL	−41.4	9.61

**Figure 11.**
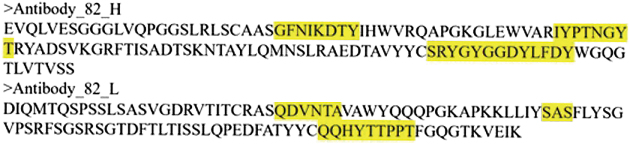
Variable region sequences and complementarity-determining region (CDR) delineation of antibody_82 heavy chain (HC) and light chain (LC).

**Figure 12.**
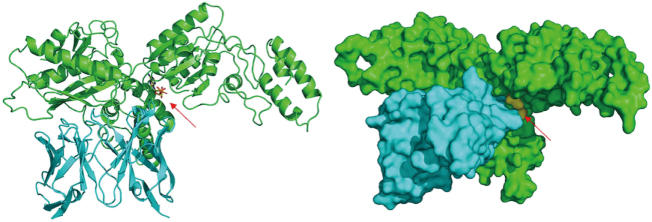
Schematic diagram of antibody-antigen molecular docking results.

We initially performed molecular docking of the antibody-target protein complex using the HDock program. Figure 11 depicts the spatial distribution of the CDRs of both the HC and LC. In Figure 12, the antibody is illustrated in blue, the target protein is illustrated in green, and the ADP molecule is represented as a yellow sphere, as indicated by the arrow. Structural analysis revealed that the antibody binds stably to the target protein, effectively blocking ADP binding and inactivating ATPase activity. This, in turn, disrupts the protease’s ability to unfold and degrade substrates.

**Figure 13.**
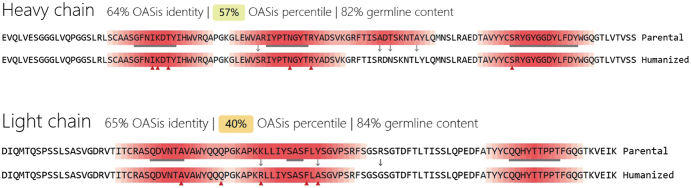
Comparison of antibody sequences before and after humanization.

**Figure 14.**
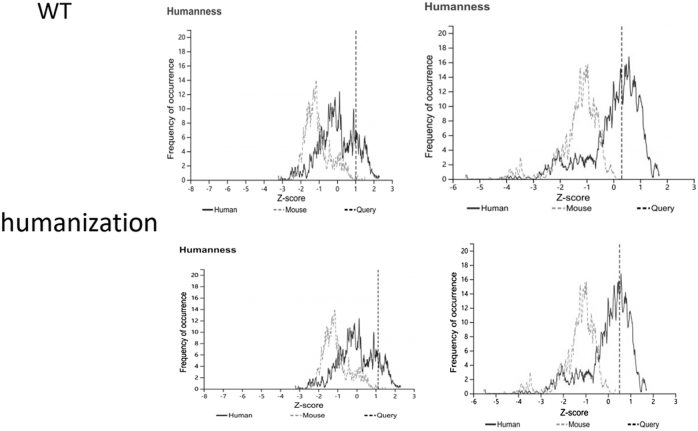
Humanization score of antibody sequences before and after humanization.

Subsequently, we employed MDs simulations to evaluate the binding characteristics of the antibody–antigen complex. The initial binding free energy was calculated to be 41.4 kcal/mol (Table 2). Energy decomposition analysis of the CDRs identified several residues that contributed unfavorably to binding (Table 3). Based on these findings, we conducted virtual saturation mutagenesis screening utilizing the CHARMM force field. Table 10 lists the most effective mutation combinations. After considering mutational effects, hydrophobicity, and developability, a triple mutant comprising HC_Lys30Glu, HC_Asp104Trp, and LC_Thr95Trp (highlighted in green in Table 4) was selected. The structure of the mutant was constructed using PyMOL, and upon optimization through MDs, the binding free energy was recalculated, demonstrating a significant improvement to 78.87 kcal/mol (Table 5). Next, the mutant antibody underwent further humanization, resulting in an increase in germline gene homology from 81% to 83% (Table 6). The humanized sequence is illustrated in Figure 13. As shown in Figure 14, the dashed line represents the *Z*-score distribution of the mouse-derived antibodies, while the solid line corresponds to the *Z*-score distribution of the human-derived antibodies. The vertical line indicates Antibody 82-M1. Following humanization, the scores of both the HC and LC shifted further to the right, indicating a greater degree of humanization. A developability assessment confirmed that all key physicochemical parameters of the antibody fall within acceptable ranges (Table 7). T-cell epitope prediction across 27 common HLA alleles indicated a limited immunogenicity risk, primarily localized within the HC_FR3 and LC_CDR2 regions (Table 8), suggesting an overall favorable safety profile. In summary, through systematic computational design and multi-dimensional optimization, Antibody_82-M1 demonstrates high target-binding affinity, low immunogenicity, and promising developability, indicating a strong potential for further preclinical development.

**Table 3:** Energy contribution breakdown of key residues in CDR regions.

region	Residue	van der Waals	Electrostatic	Polar solvation	Non-polar Solv.	TOTAL
H1	L:B:LYS:30	−0.39981	19.056095	−17.15157	−0.094436	1.4102784
	L:B:ASP:31	−0.537524	−14.485	16.013905	−0.122846	0.8685349
H2	L:B:ASN:55	−0.291095	0.0435238	0.6102381	−0.039199	0.3234682
H3	L:B:ARG:98	−0.22919	4.2685238	−3.293143	−0.028009	0.7181811
	L:B:GLY:100	−0.196619	0.5217143	0.081381	−0.000819	0.4056568
	L:B:ASP:104	−1.386619	7.6828095	−4.534619	−0.267786	1.4937853
	L:B:ASP:108	−0.245857	−0.585762	1.5204286	−0.032488	0.6563218
L3	L:C:THR:95	−0.600524	−0.869476	1.9472857	−0.091828	0.3854579

**Table 4:** Summary of optimal mutation sites from virtual saturation mutagenesis.

Region	Residue	Mutation	ΔG
H1	**L:B:LYS:30**	**Glu**	**−0.38**
	L:B:ASP:31	Glu	−0.17
H2	L:B:ASN:55	Arg	−0.32
H3	L:B:ARG:98	Glu	−0.18
	L:B:GLY:100	Trp	−2.01
	**L:B:ASP:104**	**Trp**	**−2.05**
	L:B:ASP:108	Trp	−0.3
L3	**L:C:THR:95**	**Trp**	**−2.46**

**Table 5:** Binding free energy results for affinity maturation mutants with the target.

Energy component	Average	SD
ΔVDWAALS	−93.76	12.31
AEEL	−437.56	82.97
ΔEGB	466.42	93.92
ΔESURF	−13.96	2.23
ΔGGAS	−531.32	74.69
ΔGSOLV	452.45	89.88
ΔTOTAL	−78.87	12.61

**Table 6:** Comparison before and after humanization design.

OASis identity		OASis percentile		Germline content		Germlines		Humanizing mutations	
Before	After	Before	After	Before	After	VH	VL	VH	VL
58%	65%	29%	53%	81%	83%	GHV3-66*01	IGKV1-39*01	4	3

**Table 7:** Antibody developability analysis results.

Metric	Value and flag color
Total CDR length	47
CDR Vicinity PSH Score (Kyte & Doolittle)	116.0461
CDR Vicinity PPC Score	0.0391
CDR Vicinity PNC Score	1.1695
SFvCSP Score	0

**Table 8:** Affinity fitting results.

Seq #	Peptide	Start	End	Region	Allele	Median binding percentile	Netmhcsiipan_el core	Netmhcsiipan_el score	Netmhcsiipan_el percentile
1	NSKNTLYLQMNNSLRAEDT	74	91	HC_FR3	HLA-DRB1*04:01	0.17	YLQMNSLRA	0.898772	0.17
2	APKRLLIYSASFLASG	43	58	LC_CDR2	HLA-DRB1*15:01.	0.2	LLIYSASFL	0.887071	0.2

# This naming rule is a standard established by the ImMunoGeneTics/Human Leukocyte Antigen (IMGT/HLA) database, ensuring the uniqueness and standardization of the naming of HLA alleles.

**Table 10:** Fitted parameters for LONP1 enzymatic inhibition by Antibody_82-M1.

	IC50	Top (Rmax)
mAb	0.1006	99.35%
Isotype control	-	-

### The antibody exhibits excellent physicochemical and functional properties in in vitro

**Figure 15.**
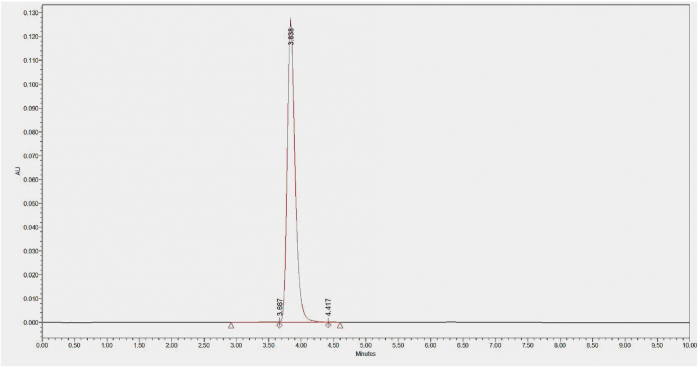
Antibody purity analysis—SEC-HPLC chromatogram.

**Figure 16.**
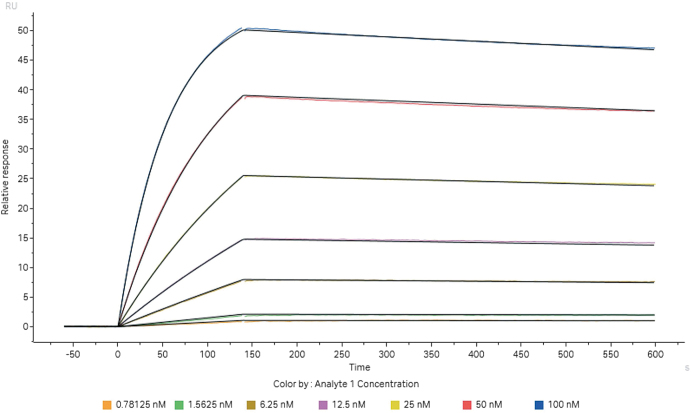
Surface plasmon resonance (SPR) sensorgram.

Furthermore, we evaluated the biological properties of the optimized Antibody_82-M1 through *in vitro* experiments. The antibody was expressed via transient transfection in HEK293 cells and purified in a single step using Protein A affinity chromatography, yielding high purity: SEC-HPLC indicated a main peak proportion of >99% (Fig. 15), and CE-SDS analysis showed a main peak accounting for 98.48% (Table 9). The interaction between Antibody_82-M1 and the LONP1 antigen was analyzed by biolayer interferometry. The experimental sensorgram is shown in Figure 16, and the fitted affinity results are summarized in Table 9. The measured association rate constant (kₐ) was 1.82 × 10^5^ M^−1^s^−1^, the dissociation rate constant (kₑ) was 1.51 × 10^−4^ s^−1^, and the equilibrium dissociation constant (K_D) was 8.29 × 10^−10^ M, indicating a potent sub-nanomolar level of binding. This affinity is significantly superior to the micromolar-level binding observed with the CDDO-Me small molecule inhibitor^[15]^.

**Table 9:** Antibody purity analysis – CE-SDS electrophoretogram.

Peak	Name	Time	RMT	Height	Raw Area	Area	%Total	%Area	Width	S/N	Baseline	Resolution
1	IS	693.2	1.000	2.3	159.4	233.6			25.7	284.0	0.6	
2		1025.3	1.479	0.1	4.1	4.0	0.17		17.6	14.2	0.6	15.15
3		1550.7	2.237	0.2	9.9	6.4	0.27		29.2	19.3	0.6	15.89
4		1798.3	2.590	0.2	12.2	6.8	0.28		36.5	19.5	0.7	4.42
5		1925.7	2.778	0.5	37.4	19.4	0.81		38.2	63.0	0.8	1.94
6		2003.6	2.891	41.2	4773’8	2366.6	98.48		53.2	4996.7	0.9	1.23

**Figure 17.**
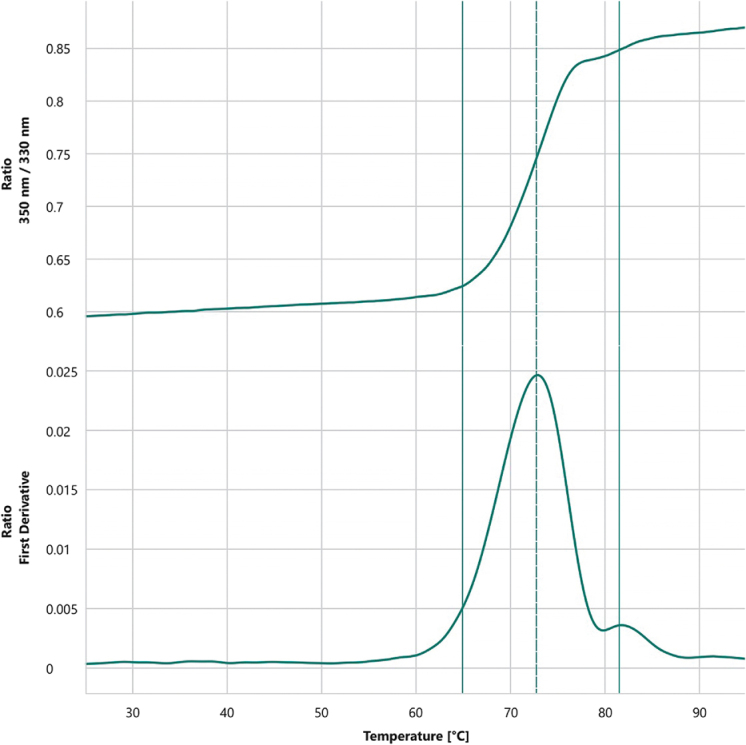
Differential scanning fluorimetry (DSF) thermal stability curve.

**Figure 18.**
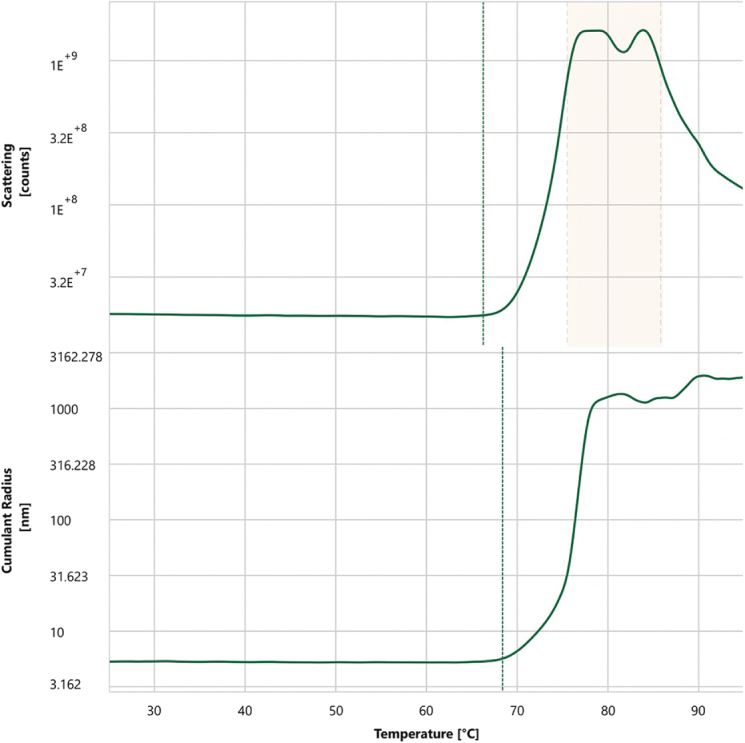
Colloidal stability analysis by dynamic light scattering (DLS) and static light scattering (SLS).

**Figure 19.**
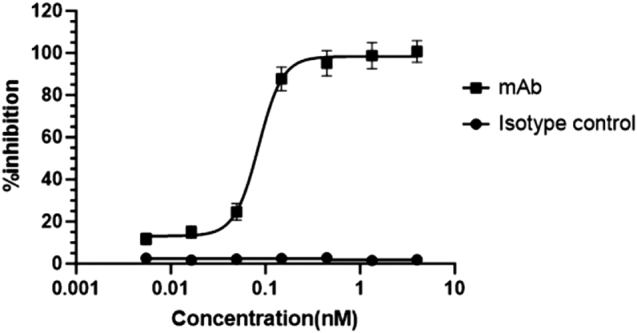
Concentration-dependent inhibition of LONP1 proteolytic activity by Antibody_82-M1.

Thermal stability analysis revealed that Antibody_82 has two melting temperatures (*T*_m_^1^ = 72.8°C, *T*_m_^2^ = 81.6°C) and an onset denaturation temperature (*T*_onset_) of 65°C, suggesting high conformational stability (Fig. 17). In terms of colloidal stability, the scattering temperature (*T*_s_c_atterin_g) was 66.4°C, and the *T*_si_Z_e_ (T_Cumulant Radius) was 68.5°C, demonstrating the antibody’s strong resistance to aggregation even under elevated temperatures and indicating favorable developability potential (Fig. 18). Subsequent *in vitro* enzymatic assays evaluating the antibody’s effect on LONP1 protease activity revealed potent inhibition, with an IC_50_ as low as 0.1006 nM and a maximal inhibition (*R*_max_) reaching 99.35% (Table 10). To rule out non-specific effects, an isotype control was included in parallel, which showed no significant inhibition of LONP1 enzymatic activity (Fig. 19).

In summary, Antibody_82-M1 demonstrates excellent characteristics in terms of expression purity, antigen-binding affinity, thermal and colloidal stability, and potent enzyme inhibition activity, underscoring its strong therapeutic potential.

## Discussion

Research trend analysis reveals a notable increase in the number of publications in the field of prostate cancer and mitochondria in recent years, with particularly prominent growth during the period from 2015 to 2017. This growth reflects the rapid development of scientific activities in this domain and its growing academic appeal. Several factors may have contributed to this trend: first, population aging has led to rising incidence rates of prostate cancer, thereby stimulating increased research attention on the disease; second, continuous advancements in biomolecular technologies have provided powerful tools for in-depth investigation of mitochondria’s role in prostate cancer pathogenesis and progression; furthermore, improved public awareness of prostate cancer has also driven growing research demand and social concern in this field^[29]^.

From the perspective of national performance, the extensive international collaboration network, particularly the substantial involvement of the two major research powers – the United States and China – further underscores the significant research value and development potential of mitochondria in prostate cancer studies.

In this study, we employed an integrated approach proceeding from dual bibliometric guidance, through research intelligent agent screening, to bioinformatics support for the selection of LONP1 as the target. Using GeoBiologics, we designed an inhibitory antibody to block its ATP-binding site, thereby suppressing its protease activity. While AI platforms currently enable antibody design, the process still requires substantial human supervision and optimization. However, with increasing computational power and iterative improvements in design frameworks, the development of fully autonomous antibody design agents is highly promising in the near future.

Most approved antibody drugs primarily target membrane proteins, and their clinical limitations are increasingly recognized. These include the inherent conformational dynamics of membrane proteins, low target selectivity due to high intra-family homology, complex negative feedback regulation, redundancy and crosstalk among signaling pathways, as well as acquired resistance during treatment and off-target toxicity resulting from broad tissue distribution^[30,31]^.

Our investigation into LONP1 as an intracellular target for prostate cancer represents a forward-looking breakthrough endeavor. Several innovative delivery strategies – such as metal-phenolic network nanoparticles^[32]^, cubosome-exosome fusion systems^[33]^, and histidine-rich cell-penetrating peptides^[34]^ – show promise for efficient intracellular antibody delivery. The deepening integration of artificial intelligence and smart material design is expected to facilitate more precise and efficient antibody-carrier co-design, accelerating the development of a new generation of targeted therapies characterized by high intracellular delivery efficiency, low toxicity, and reduced cost.

Furthermore, our literature search identified 152 studies on mitochondrial-targeted therapies for prostate cancer, 29 of which involved combination strategies aimed at enhancing efficacy and overcoming the limitations of monotherapy through synergistic multi-mechanism approaches^[35]^. Reported combination regimens include targeted antibodies with hormone therapy (e.g., abiraterone and enzalutamide), chemotherapy (e.g., docetaxel), radiotherapy (e.g., external beam radiation therapy), anti-angiogenic therapy (e.g., olaratumab and anlotinib), bone metastasis treatment (e.g., denosumab), and PARP inhibitors (e.g., olaparib)^[36–39]^. Therefore, in subsequent research and clinical translation of our developed LONP1 antibody, it is essential to actively explore combination strategies with the aforementioned treatments to construct multimodal intervention approaches, ultimately improving cure rates and quality of life for prostate cancer patients.

Finally, the emergence of AI scientists like Biomni and Kosmos is driving a profound transformation in research paradigms. While numerous AI agents now assist in target screening, they primarily rely on public databases, self-generated sequencing data, and literature mining. This study, initiating from bibliometric analysis and systematically screening targets through bioinformatic approaches, offers a novel perspective for drug target discovery and provides a referential pathway for scientific topic selection. AI researchers can build upon the methodology presented here by integrating macro-analytical tools like bibliometrics into their large language model architectures, or incorporating them as components of evaluation and inspiration mechanisms. This integration holds promise for further optimizing research question generation, direction assessment, and resource allocation, thereby advancing life sciences research into a more intelligent, open, and efficient new era.

## Conclusion

The aforementioned research provides novel insights into the treatment of CRPC. Although mitochondrial-targeted therapy shows promising potential in the management of CRPC, fully realizing its benefits will require addressing existing challenges through rigorous scientific investigation and well-designed clinical trials. With continuous advancements in science and technology, it is anticipated that further breakthrough discoveries will emerge, offering more effective and safer therapeutic options for patients with CRPC.

## Data Availability

Publicly available.

## Research registration unique identifying number (UIN)

This study did not involve human *subjects.*
